# Diuretics vs. placebo for postpartum blood pressure control in preeclampsia (DIUPRE): a randomized clinical trial

**DOI:** 10.1186/s12978-015-0057-0

**Published:** 2015-08-05

**Authors:** Telma Cursino, Leila Katz, Isabela Coutinho, Melania Amorim

**Affiliations:** Instituto de Medicina Integral Prof. Fernando Figueira, Recife, PE Brazil; Department of Obstetrics and Gynecology, Federal University of Campina Grande, Campina Grande, PB Brazil

**Keywords:** Preeclampsia, Very high blood pressure, Diuretics, Randomized controlled trial

## Abstract

**Background:**

Hypertension affects about 10 % of pregnancies and is responsible for both maternal and neonatal devastating complications such as eclampsia, HELLP syndrome, prematurity and maternal and neonatal death. The resolution of the disease occurs in the first twelve weeks postpartum. The behavior of blood pressure and occurrence of very high blood pressure episodes among women with severe preeclampsia is related to remodeling of the dynamics of body fluids with consequent increase in intravascular volume. The persistence of hypertension in critical levels results in increased puerperal morbidity.

**Objectives:**

To evaluate the effectiveness of furosemide in accelerating blood pressure recovery among women with severe preeclampsia.

**Methods/design:**

A triple-masked placebo controlled clinical trial, will be conducted including 120 postpartum women with severe preeclampsia, after eclampsia prophylaxis with magnesium sulfate and with adequate diuresis. Women with chronic hypertension and users of diuretics will be deleted. Informed consent will be obtained from all participants. Patients will be randomized to receive furosemide (40 mg orally every twenty four hours) or placebo. The variables are systolic and diastolic blood pressure, frequency of hypertensive crises, need for maintenance of antihypertensive therapy, number of antihypertensive agents used to control blood pressure, urine output, length of hospital stay, adverse effects and maternal complications. This study was approved by the Research Ethics Committee in humans of our institution. All participants will be duly informed about the aims of the project and will be included only if they agree to participate voluntarily, signing the informed consent.

**Trial registration:**

This study was registered on Clinical Trials.gov under the number NCT02163655. (http://clinicaltrials.gov/show/NCT02163655).

## Introduction

Hypertension in pregnancy continues to represent a real public health issue. Affecting 10 % of all pregnancies [1], hypertension is the first cause of maternal death in Brazil http://inseer.ibict.br/rbsp/index.php/rbsp/article/viewFile/474/pdf_150 and the second cause in the world [2, 3]. Worldwide, more than 99 % of cases of maternal deaths related to hypertension occur in low-income countries, reflecting not only a higher incidence as the lack of prenatal care and adequate treatment of obstetric emergencies [2]. Moreover, it is an important potentially life-threatening condition of life and cause of maternal near miss [4], along with increased perinatal morbidity and mortality [2]. It is also associated with long-term complications, with increased risk of cardiovascular disease in affected women [5, 6].

Among hypertensive disorders complicating pregnancy, pre-eclampsia and eclampsia represent the most severe conditions [7, 8]. Associated maternal risks include abruptio placentae, convulsions, acute pulmonary edema, convulsions, acute kidney injury and death. Perinatal risks are due to the reduction in the utero-placental blood flow and are represented by fetal growth restriction, hypoxia and fetal death, prematurity and its complications, among which the most important is the respiratory distress syndrome of the newborn [9].

It is well known that magnesium sulfate is the most effective and safe drug for preventing and treating eclamptic seizures and should be administered to all women in whom there is concern about eclampsia or eclampsia risk [10–14]. Magnesium sulfate significantly reduces maternal death when used to treat eclampsia and is a real "evidence-based solution" http://www.engenderhealth.org/files/pubs/maternal-health/engenderhealth-eclampsia-report.pdf. The only definitive treatment is delivery, and the resolution of pregnancy is indicated in all cases of eclampsia regardless of gestational age [9] in cases of preeclampsia at ≥ 34 weeks [9, 15, 16] and mild preeclampsia cases without other complications at ≥ 37 weeks [17].

However, even after birth and with appropriate treatment, concern about the complications of disease persist, since this condition can take days or weeks to resolve. In the postnatal period, with the return of liquid from the interstitial and extravascular compartment to the intravascular, very high blood pressure episodes are common and the persistence of hypertension in critical levels results in increased puerperal morbidity. Thus, blood pressure levels may continue to rise, which most often occurs in the first five days after birth, reaching a peak between three to six days after delivery [18]. New onset postpartum hypertension and preeclampsia can occur, with a frequency ranging from 0,3 %-27,5 % [19]. Complications of severe hypertension include stroke, eclampsia, congestive heart failure, acute pulmonary edema, renal failure and death [20].

Besides the mobilization of liquid from the interstiticial and intravascular to extravascular space corresponding to six to eight liters of the total body water, there is also a return of 950 mEq of total body sodium accumulated during pregnancy. It is observed increased urinary sodium excretion between three and five days after birth [21] possibly due to the increase of atrial natriuretic peptide in the first week after delivery, which has a role in natriuresis and entails inhibition of aldosterone, angiotensin II and vasopressin [22]. Several approaches have been proposed to accelerate the postpartum maternal recovery, but the effectiveness of these approaches as well as the ideal antihypertensive treatment in the postpartum period, remain to be established. Furosemide use has been proposed with this goal. Patients with preeclampsia and eclampsia may experience persistent hypertension after delivery, in response to the mobilization of interstitial and extravascular fluid into the intravascular space, the excess of total body water and inadequate secretion of sodium due to reduced glomerular filtration [23]. This is an important rationale for furosemide therapy since by its mechanism of action, this loop diuretic can act in patients with fluid overload, eliminating the intravascular fluid that has been mobilized in the postpartum period and thereby reducing blood volume and blood pressure and also reducing the need of antihypertensive therapy [23]. This therapy is logical when considering the pathophysiology of postpartum hypertension, with the objective of maintain a low central venous pressure and pulmonary capillary wedge pressures, raising colloid osmotic pressure and preventing the development of pulmonary edema and congestive heart failure [24].

In the systematic Cochrane Review that evaluated prevention and treatment of postpartum hypertension were included nine randomized clinical trials, of which two trials (282 women included) assessed postnatal furosemide at a dosage of 20-40 mg orally daily), compared with placebo or no treatment [25]. One trial added potassium supplementation (20 mEq orally daily) [23] and in the other [26] women at “high risk” of eclampsia also received magnesium sulfate. None of the studies reported the mean and standard deviation of the baseline blood pressure but one trial [26] reported a greater decrease in blood pressure in the group receiving furosemide compared to placebo. In the group receiving furosemide it was reported a change of blood pressure on days one, three and seven after delivery (i.e., −6.5 mmHg (furosemide) versus −3.5 (placebo) on day one, −10.6 (furosemide) versus −9.75 (placebo) on day three, and −11.5 (furosemide) versus −7.8 (placebo) on day seven). In the other trial [23] including 264 women, only postpartum patients with severe preeclampsia (*n* = 70) who received furosemide compared with controls had significantly lower systolic blood pressure by postpartum day 2 (142 +/− 13 mmHg compared with 153+/−19 mmHg, *P* < .004) and required less antihypertensive therapy during hospitalization (14 % compared with 26 %, *p* = 0.371) and at discharge (6 % compared with 26 %, *P* = 0.045). Follow-up ranged from day 10 postpartum [23] to six weeks postpartum [26].

The reviewers conclude that reliable data are not available to guide management of women with postpartum hypertension. For women with pre-eclampsia, postnatal furosemide may decrease the need for postnatal antihypertensive therapy in hospital, but more data are needed on substantive outcomes before this practice can be recommended. Notwithstanding, the optimal management of postpartum hypertension is not yet well established.

Thus, to determine the potential benefit of using diuretics in women with preeclampsia is an important issue in modern obstetrics. This study will be carried out with the objective of determining the effectiveness of furosemide to accelerate the recovery of hypertension in postpartum patients with severe preeclampsia.

## Objectives and hypothesis

The overall objective is to determine the effectiveness of furosemide to accelerate the recovery of hypertension in postpartum patients with severe preeclampsia in a randomized clinical trial.

The hypothesis is that mean daily average systolic, diastolic and mean arterial pressure (MAP) and the frequency of very high blood pressure episodes when furosemide is administered is lower.

### Specific objectives

In patients with severe preeclampsia receiving furosemide or placebo in postpartum, compare:

## Primary outcomes

- Daily average of systolic, diastolic and mean arterial pressure (MAP);
- Heart rate;
- Frequency of very high blood pressure episodes.

### Secondary maternal outcomes

- Control of blood pressure;
- Frequency of use of antihypertensive agent for the treatment of hypertensive crisis;
- Need for maintenance of antihypertensive therapy for blood pressure control;
- Number of antihypertensive agents used to control blood pressure at hospital discharge;
- Time until blood pressure control;
- Daily urine output;
- Reduction of edema;
- Length of hospital stay;
- Adverse effects: hypokalemia, polydipsia, headaches, mental confusion, muscle aches, tetany, muscle weakness, heart rhythm disturbances and gastrointestinal symptoms;
- Frequency of maternal complications: imminent eclampsia, eclampsia, infection, bleeding manifestations, shock and maternal death.

### Main hypothesis

In women randomized to postpartum oral furosemide, in comparison to placebo:

- Daily averages of systolic, diastolic and mean arterial pressure (MAP) are lower, heart rate is the same and the frequency of very high blood pressure episodes is lower.
- Control of blood pressure is obtained earlier;
- Frequency of use of antihypertensive agent for the treatment of hypertensive crisis is lower, as well as the need for maintenance of antihypertensive therapy for blood pressure control;
- Fewer antihypertensive agents are needed in order to control blood pressure at hospital discharge;
- Time until blood pressure control is lower;
- Daily urine output is higher;
- Reduction of edema is obtained in less time;
- Length of hospital stay is reduced.

## Methods/design

### Study design

The present study is a triple masked randomized controlled trial.

### Study population and location

The study population will comprehend all women hospitalized in IMIP during the data collection period with the diagnosis of preeclampsia/eclampsia.

### Eligibility criteria

Postpartum women diagnosed with severe preeclampsia, after completion of therapy with magnesium sulfate, with spontaneous diuresis and greater than 50 ml/h. Women with superimposed preeclampsia, or that used diuretics before admission to the intensive care unit (ICU), with acute or chronic kidney disease, diabetes mellitus; collagen disease; sickle cell anemia, patients with hemodynamic instability or with hypokalemia (K < 3.0 mEq/L) or patients with contraindications to the use of furosemide or unable to consent were excluded.

### Procedures for selecting participants and randomization

Eligible patients in labor will be invited to participate and those who agree and sign the informed consent form will be included in the study and then will be allocated to either using furosemide or placebo according to a random list of numbers generated by the Random Allocation Software Ispharan Iran, version 1.0. This list of randomization will be provided by the statistician to the pharmacist who will be responsible for preparing the packages containing either the furosemide or placebo, both in an identical presentation, with the identification number of list labeled. This procedure will be followed in order to guarantee the concealment of allocation of patients in both arms. Both patients and medical staff should be blind of the intervention condition in each case. Study medication and placebo, after delivered by the pharmaceutical industry to the pharmacy, will be packed as previously described and according the random list. The pharmacy will be responsible for sending sets to the ICU were the nurse in charge will keep all the packages until the administration of the drug to the patient according to the patients’ enrollment. The subjects will receive the study medication every 24 hours for a maximum of five days.

During the observation period the women will be subject to strict control of blood pressure, urine output (diuresis evaluated spontaneous or urinary catheter) and weight control. The routine management adopted at the hospital will be followed and other hypertensive agents will be initiated and chose according to the attending clinician's discretion.

### Sample size calculation

The calculation of sample size was done in the online public domain software version 2.3 Openepi (Atlanta, GA). Were used as reference data found in clinical trial using furosemide versus placebo, in which they found an average systolic blood pressure of 142 mmHg and a standard deviation of 13 mmHg and 153 mmHg and a standard deviation of 19 mmHg, respectively. For a power of 90 % and a significance level of 5 %, 94 patients would be needed in both groups to highlight this difference. In anticipation of possible post-randomization losses added up to total 20 % of patients, resulting in 120 for the two groups.

### Variables

#### Independent variable:

Furosemide or placebo.

### Dependent variables

Daily maximum systolic blood pressure; daily maximum diastolic blood pressure; maximum average daily blood pressure; heart rate; frequency of very high blood pressure episodes; control of blood pressure; need for maintenance of antihypertensive therapy for blood pressure control; number of antihypertensive agents used to control blood pressure; time required for recovery of blood pressure; urinary output; length of hospital stay; adverse effects (polydipsia, headache, confusion, muscle aches, tetany, muscle weakness, heart rhythm disturbances, gastrointestinal symptoms); puerperal complications during hospitalization (acute pulmonary edema, hemorrhagic manifestations, imminent eclampsia, eclampsia, maternal death, shock, persistent edema.

### Main outcomes

- Systolic blood pressure (SBP): Determined by the appearance of the first sound of Korotkoff. Continuous quantitative variable, expressed in millimeters of mercury (mmHg).
- Diastolic Blood Pressure (DBP): Determined by the appearance of the fifth Korotkoff sound. Continuous quantitative variable, expressed in millimeters of mercury (mmHg).
- Mean arterial pressure (MAP): The value of mean arterial pressure in mmHg. Will be calculated by the formula: systolic blood pressure (SBP) plus twice the diastolic blood pressure (DBP) divided by three, being represented by (+2PAD SBP)/3. Continuous quantitative variable, expressed in millimeters of mercury (mmHg).
- Heart rate: Refers to the number of heart beats per minute, measured at least four times daily.
- Frequency of very high blood episodes: Defined as the daily number of very high blood pressure episodes, defined as Systolic blood pressure ≥ 180 mmHg or Diastolic blood pressure ≥ 110 mmHg.
- Control of blood pressure: Defined < 140 mmHg of systolic blood pressure and < 90 mmHg of diastolic blood pressure at all measurements (at least four) for a minimum 24-hour.
- Need for maintenance of antihypertensive therapy for blood pressure control: Use of anti-hypertensive agents for blood pressure control.
- Number of antihypertensive agents used to control blood pressure: Number of antihypertensive agents used to control blood pressure.
- Time required for recovery of blood pressure: Time to achieve control of blood pressure expressed in whole numbers.
- Hospital stay: For time elapsed between the beginning of inpatient treatment with furosemide or placebo until discharge.
- Daily urine output: Defined as the average amount of urine excreted, expressed in milliliters (ml), collected by probe or spontaneous voiding during hospitalization.

### Data collection procedures

#### Data collection

For data collection, a pre-coded standard form will be used for data entry on the computer. After identifying the patients who are according to the eligibility criteria, using a specific check list, and who agree to participate in the study and sign the informed consent form, information will be collected and filled in the form. The checklist, as well as the form will be completed by the researchers.

Upon completion, the forms will be rigorously reviewed by the researchers to cross-check the information collected with the information contained in the records. Procedures for quality control, such as reviewing the completed forms and check manually typing will be adopted. A first quality control of data collection should be done before and during the typing of electronic records, to identify possible inconsistencies in the data researcher. The second quality control will check the compatibility between the physical archived records and data contained in electronic forms. The data will be entered in a specific database, created using the Epi Info statistical program 7.1.4 or an updated version available at this time. Each month the database will be reviewed by the principal investigator, getting listing of variables and correcting any inconsistencies or missing information from the query to the forms.

### Data analysis plan

The data analysis will be performed using the public domain software Epi Info version 7.1.4 (Centers for Disease Control and Prevention, Atlanta, GA), or the newest available version at the time of analysis) under the intention to treat principle.

**Fig. 1:**
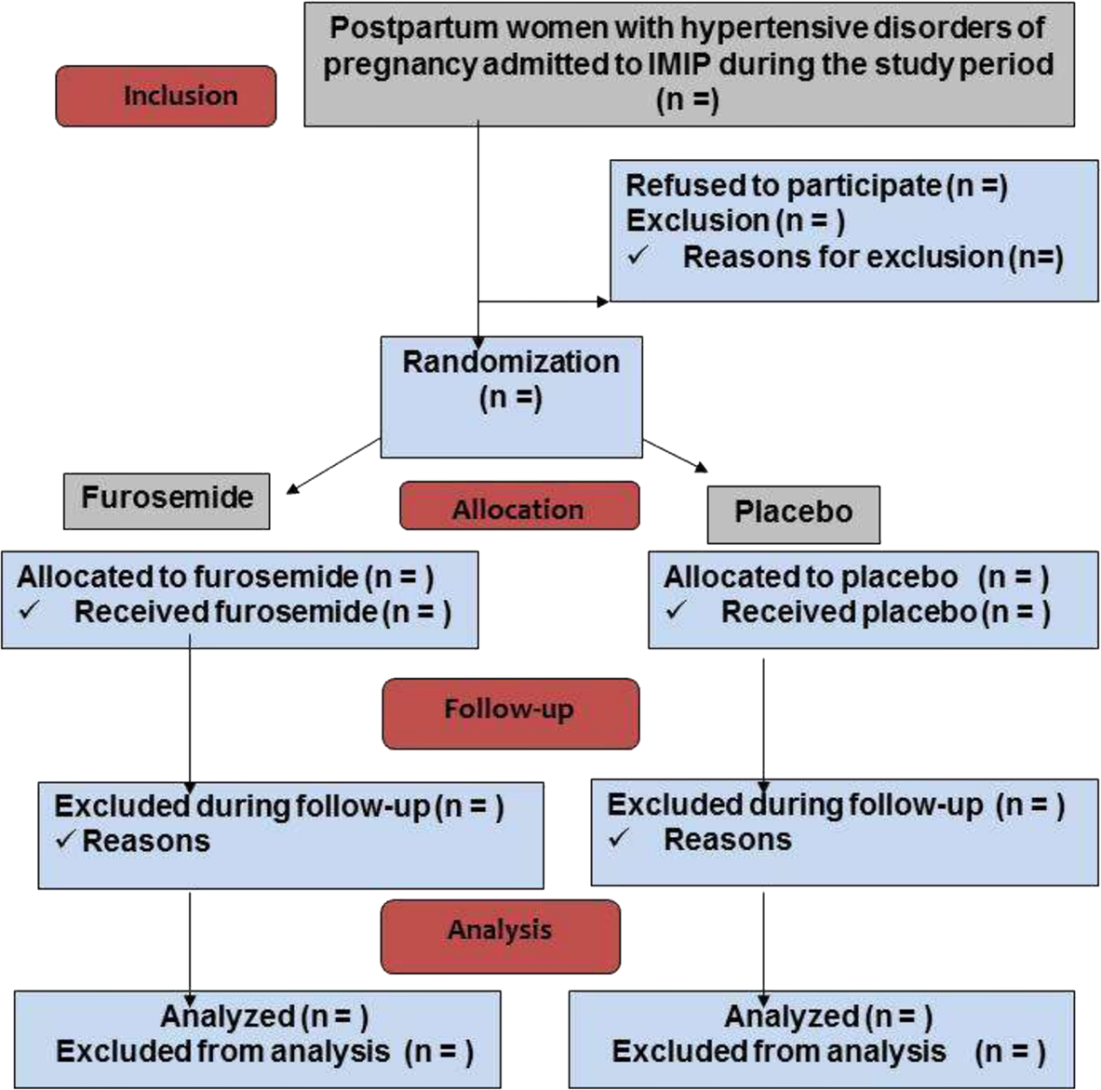
Study design and population (CONSORT, 2010) [27]

The statistician will remain blind to the meaning of the Groups A or B to which patients are allocated until the tables and the analysis concluded. The approach for analysis will be that showed in Fig. 1 using an intention-to-treat strategy and following the correspondent recommendations from the CONSORT statement [11].

The characteristics of the participants in each group will be compared with Student’s *t* test for continuous variables with normal distribution and Mann–Whitney U test for discrete and ordinal variables or those with non-normal distribution. Categorical variables will be compared with Pearson’s χ [2] test or Fisher’s exact test, as appropriate. P values for all tests will be two tailed at a 5 % level of significance.

Risk ratios and their 95 % confidence intervals will be calculated as a measure of relative risk. The number needed to treat (NNT) and its 95 % confidence interval will be calculated for the outcomes in which a beneficial effect of using furosemide is achieved, using the EBM calculator [http://moosenose.com/EBCalculator.htm]; in case of adverse effects the number needed to harm (NNH) and its 95 % confidence will also be calculated.

### Quality control

The researchers will maintain a record of problems occurred during the study and any doubt should be solved with the Steering Committee.

### Ethical issues

The original protocol of this research proposal has already obtained approval of the local Institutional Review Board from the coordinating center (IMIP, Recife, Brazil), and of the National Committee for Ethics in Research (CONEP) of the Brazilian Ministry of Health, under the number 3957. The protocol also was published in the Clinical Trials Register under the number NCT02163655. (https://clinicaltrials.gov/ct2/show/NCT02163655).

Patients will only be included if they agree to participate and sign the informed consent. All principles related to research in human beings established by the Brazilian National Health Council according to the Declaration of Helsinki will be followed. The confidentiality on women’s data and medical care will be ensured regardless of whether they participate in the study or not.

## Discussion

### Technical and scientific contributions of the study

Treatment of patients with preeclampsia and eclampsia still represents a challenge in the postpartum period, since the pressure levels may continue to rise, which increases morbidity, mortality and length of hospital stay. There is a rationale for the use of furosemide for the purpose to reduce these complications since this drug eliminates excess intravascular volume resulting from the mobilization of liquid which was previously in the interstitial and extravascular space. However, evidence from randomized clinical trials is scarce and insufficient for a recommendation to incorporate the use of furosemide in daily clinical practice.

Furosemide, a loop diuretic, is a cheap drug, safe, widely available and considered level 1 for use during breastfeeding, especially if used for shorter periods. Long-term treatment may inhibit lactation but is not the case of this proposed therapy, using low dose of only five days after birth. If it is demonstrated its effectiveness in reducing blood pressure levels, the need for antihypertensive drugs and shortening of hospitalization, significant benefits can be obtained for women with pre-eclampsia and the health system. With the reduction in blood pressure and the elimination of excess intravascular, the incidence of acute pulmonary edema and other diseases resulting from fluid overload can be decreased. The reduced need for antihypertensive drugs, beyond avoiding side effects for women, can along with the reduction of hospital days, lead to substantial savings for the health system.

## Abbreviations

IMIP: Instituto de Medicina Integral Prof. Fernando Figueira
SBP: Systolic blood pressure
BDP: Diastolic blood pressure
MAP: Mean blood pressure
NNH: Number needed to harm
NNT: Number needed to treat
CONEP: National Committee for Ethics in Research of the Brazilian Ministry of Health
ICU: Intensive care unit
RCT: Randomized controlled trial
